# Quantifying kinematics of purposeful movements to real, imagined, or absent functional objects: Implications for modelling trajectories for robot-assisted ADL tasks**

**DOI:** 10.1186/1743-0003-4-7

**Published:** 2007-03-23

**Authors:** Kimberly J Wisneski, Michelle J Johnson

**Affiliations:** 1 Marquette University, Dept. of Biomedical Engineering, Olin Engineering Center, Milwaukee, WI USA; 2 Medical College of Wisconsin, Dept. of Physical Medicine & Rehabilitation, 9200 W. Wisconsin Ave, Milwaukee, WI 53226, USA; 3 Clement J. Zablocki VA, Dept. of Physical Medicine & Rehabilitation, 5000 National Ave, Milwaukee, WI, USA; 4 The Rehabilitation Robotics Research and Design Lab, 5000 National Ave, Milwaukee, WI, USA

## Abstract

**Background:**

Robotic therapy is at the forefront of stroke rehabilitation. The Activities of Daily Living Exercise Robot (ADLER) was developed to improve carryover of gains after training by combining the benefits of Activities of Daily Living (ADL) training (motivation and functional task practice with real objects), with the benefits of robot mediated therapy (repeatability and reliability). In combining these two therapy techniques, we seek to develop a new model for trajectory generation that will support functional movements to real objects during robot training. We studied natural movements to real objects and report on how initial reaching movements are affected by real objects and how these movements deviate from the straight line paths predicted by the minimum jerk model, typically used to generate trajectories in robot training environments. We highlight key issues that to be considered in modelling natural trajectories.

**Methods:**

Movement data was collected as eight normal subjects completed ADLs such as drinking and eating. Three conditions were considered: object absent, imagined, and present. This data was compared to predicted trajectories generated from implementing the minimum jerk model. The deviations in both the plane of the table (XY) and the saggital plane of torso (XZ) were examined for both reaches to a cup and to a spoon. Velocity profiles and curvature were also quantified for all trajectories.

**Results:**

We hypothesized that movements performed with functional task constraints and objects would deviate from the minimum jerk trajectory model more than those performed under imaginary or object absent conditions. Trajectory deviations from the predicted minimum jerk model for these reaches were shown to depend on three variables: object presence, object orientation, and plane of movement. When subjects completed the cup reach their movements were more curved than for the spoon reach. The object present condition for the cup reach showed more curvature than in the object imagined and absent conditions. Curvature in the XZ plane of movement was greater than curvature in the XY plane for all movements.

**Conclusion:**

The implemented minimum jerk trajectory model was not adequate for generating functional trajectories for these ADLs. The deviations caused by object affordance and functional task constraints must be accounted for in order to allow subjects to perform functional task training in robotic therapy environments. The major differences that we have highlighted include trajectory dependence on: object presence, object orientation, and the plane of movement. With the ability to practice ADLs on the ADLER environment we hope to provide patients with a therapy paradigm that will produce optimal results and recovery.

## Background

Stroke is a major cause of adult disability in the United States with over 5 million people living in the US with post stroke effects. Often stroke survivors are left with severe disability and hemiparesis that makes functioning in their daily living environment extremely challenging or impossible without complete assistance. There is a need for improved methods of rehabilitation and an increase in the rehabilitation efforts made [1,2].

The question of what is the best approach to stroke therapy is currently a topic for much debate [3,4]. The literature supports that effective therapies contain elements of repetition, intense practice, motivation, and task application [5-13]. Enriched environments, patient involvement and empowerment, and functional and purposeful tasks have been shown to increase patient motivation, recovery, and carryover of learned function to the home [3,5-22]. For example, research performed by Trombly, Wu, and colleagues showed that both normal subjects and stroke survivors reach more efficiently and accurately when functional objects were used as the reach target, i.e., when the reach was more purposeful [3,17-19]. They proposed that when objects are present (object present) as a goal the person can obtain sensory information regarding the task at hand while if there is no object available (object absent), there is no visual goal information by which to organize movement. Although further research is still needed, these results suggest that these therapeutic approaches can improve functional outcomes for the stroke patient and suggest that robot-assisted therapy may benefit by increasing its task-oriented nature.

Currently, robotic therapy environments are promising tools for stroke rehabilitation. They are capable of administering therapy under minimal supervision, at high intensity, and for long durations. The effectiveness of some of the current robotic therapy systems, such as the MIT-Manus [23], the GENTLE/s [24] and the MIME [25,26], has been tested and typically patients using them have seen faster recovery, decreased impairment, increased accuracy of movement, decreased task completion time, and smoother movements than their counterparts who received traditional therapy [2,27,28]. Despite these successes, robot therapy environments often face the same problems that conventional therapy methods face when it comes to carryover of motor gains to real life. Patients who use current robotic therapy environments show inconsistent carryover of the gains made in a therapy session to their home environment. It has been suggested that this may be because the patients are not performing 'real' tasks in the therapy environment so they are not able to map the movement kinematics that they learned in therapy to their daily tasks.

**Figure 1:**
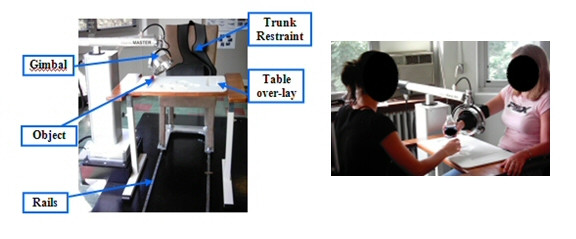
**The ADLER therapy environment**. Left: The ADLER therapy environment consisting of a chair on rails that is pulled up to an activity table. The chair has a built in trunk restraint to isolate arm movement. The patient interacts with the system by way of an orthosis that is attached to the gimbal (end effector) of ADLER. ADLER is a 6 degree of freedom (3 active, 3 passive) robot. Right: ADLER operating in a functional task environment as the subject reaches to a bottle of water.

We have set out to work towards combining the benefits of robot therapy with task-oriented therapy focused on the practice of real activities of daily living (ADLs). The move to integrate robot therapy with ADL practice is new in the field and has been addressed by few systems, including the Automated Constraint-Induced Therapy Extension (AutoCITE) system [26,27] and the MIT-MANUS [28,29]. With this goal in mind, we designed the Activities of Daily Living Exercise Robot (ADLER) therapy system [30]. The ADLER environment supports both 2-D and 3-D point-to-point reaching as well as functional task-oriented exercise involving both reaching and manipulation (Fig. 1).

To assist both reaching and/or manipulation movements in the ADLER therapy environment, trajectories must be programmed in to the robotic system. Currently the trajectories that are programmed into most robotic therapy environments are based on 5^th ^or 7^th ^order polynomials derived from the minimum jerk theory of movement assuming zero start and end velocities and accelerations and straight line movements [31,32]. Developed by Hogan and Flash [31,37-42] after observing invariant patterns in point-to-point human arm movements, the minimum jerk theory for multi joint arm movements have been shown to work well for point-to-point trajectory generation in many robotic therapy environments [33-36]. However, since we wish to not only support point-to-point movements, but also full functional movements in the ADLER environment, it is necessary for us to analyze natural movements to real life objects and determine if the current models are adequate (Fig. 1b).

In this paper, we used motion analysis tools to investigate functional movements generated during the completion of tasks such as eating and drinking. We compare these functional movements to those predicted by the minimum jerk model since this model is often used for trajectory generation during robot-assisted movements. Specifically, we investigate the motor performance of able-bodied subjects on functional eating and drinking tasks under three conditions; 'object present', 'object absent', and 'object imagined'. We fit the 5^th ^order minimum jerk model to the data and examine the differences seen in the movement under the three conditions. We hypothesized that due to the addition of functional constraints and requirements the minimum jerk trajectory model will fit the 'object absent' condition the best and the 'object present' condition the least. Only, the initial reaching event of the drink and feed tasks are analyzed and presented below. Since the minimum jerk trajectory theory was developed based upon the observations of point-to-point reaching movements. We expected that the reaching events of these tasks to produce trajectories that were most comparable to those predicted by the minimum jerk model. We discuss implications of these findings for modelling functional movements and robot mediated support of these movements.

## Methods

The study was conducted at the Human motion analysis lab at the Froedtert Hospital and Medical College of Wisconsin (MCW) and was approved by the institutional review board of MCW. We examined the data for eight able-bodied subjects who gave their consented for study. The functional movement data of subjects performing activities of daily living (ADLs) were collected using a 15-camera Vicon 524 Motion Analysis System (Vicon Motion Systems Inc.; Lake Forest, CA). The subjects were right-handed individuals ranging in age from 20 to 72 with an average age of 38 years. They were asked to perform 24 ADL tasks three times each in a random order (i.e. eating, drinking, combing hair, etc.). Although the 24 tasks consisted of both bilateral and unilateral activities, only the unilateral drinking and feeding tasks are examined in this paper. Specifically, six tasks are examined: non-dominant (ND) drink object present, ND drink object imagined, ND drink object absent, ND spoon feed object present, ND spoon feed object imagined, and ND spoon feed object absent. Table 1 lists the three task conditions as well as the events that had to be completed for each one. The initial reaching event (event 1) of these functional tasks are analyzed and then compared to a point-to-point reaching task as defined by the minimum-jerk model.

**Table 1:** Drink and Feed Tasks Conditions and Corresponding Events

**Drink**	**Object Present**	**Object Imagined**	**Object Absent**
Event 1	Reach to cup	Reach to imaginary cup	Touch center of table (approx. 10 inches from edge)
Event 2	Bring cup to mouth (drink)	Pretend to drink from imaginary cup	Touch mouth
Event 3	Return cup to table	Return imaginary cup to table	Touch center of table again
Event 4	Return to rest	Return to Rest	Return to Rest

**Feed**	**Object Present**	**Object Imagined**	**Object Absent**

Event 1	Reach to spoon	Reach to imaginary spoon	Touch ND side of table (approx. 7 inches from edge and 7 inches from midline)
Event 2	Bring spoon to bowl to scoop pudding	Pretend to scoop pudding from imaginary bowl	Touch center of table (approx 5 inches from edge)
Event 3	Bring spoon to mouth to eat pudding	Bring imaginary spoon to mouth	Touch mouth
Event 4	Return spoon to table	Return imaginary spoon to table	Touch ND side of table again
Event 5	Return to Rest	Return to Rest	Return to Rest

### The Drink Task (Present, Imagined, and Absent)

**Figure 2:**
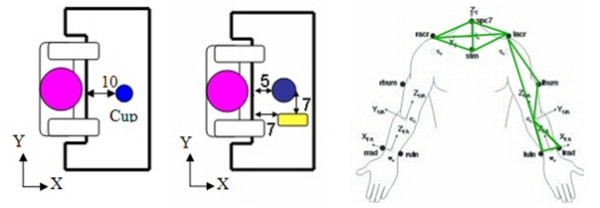
**Activity Table Set-Up & Marker Placement**. Left (a): A bird's eye view of the activity table set up for the drink task. The cup is 10 inches from the table edge along the subject's midline. Middle (b): A bird's eye view of the activity table set up for the feed task. The bowl is 5 inches from the table edge along the subject's midline and the spoon is 7 inches from the table edge and displaced 7 inches from the subject's mid line on his/her non-dominant side. Right (c): The 12 markers placed on specific bony landmarks to define 5 body segments (3 non-collinear markers per segment): right and left upper and lower arms and trunk.

Subjects were asked to complete the drink task under three conditions: 'object present', 'object imagined', and 'object absent'. First, for the 'object present' condition, the drink task consisted of the following events: reach out to cup, bring cup to mouth, take a drink, return cup, and return arm to rest, which was defined as hands pronated, palms flat on table in a designated position, and elbows at 90 degrees. The cup was placed at the subject's midline, 10 inches from the table edge (Fig. 2a). The cup used had a handle that protruded at a 135° angle. Subjects were asked to complete the task just as they would in real life. Second, in the object imagined condition, the subjects were told to imagine a functional cup and imagine the same events as were executed in the object present condition. When asked after testing, the subjects reported that they imagined a coffee mug type cup with a handle that was parallel to the cup. Finally, in the object absent or point-to-point condition, the subjects were shown a series of points to touch during the task with no reference given to the actual task itself. These points did not have a visual representation in order to eliminate any functional cues. The subjects received all tasks in random order as to eliminate the influence of performing any one condition prior to others.

### The Feed Task (Present, Imagined, and Absent)

The feed task was also performed under all three conditions. This task is used in the analysis since it differs from the drink task in both object orientation and location. The spoon used in this feed task was placed 7 inches from the table edge and 7 inches from the subject's midline on the ND side (Fig. 2b). The feed task was performed in the same three conditions as the drink task.

### Data Collection

For motion data collection subjects were instrumented with 12 reflective markers attached over specific bony landmarks using double sided hypoallergenic tape (Fig. 2c). Fifteen Vicon cameras (Vicon Motion Systems Inc.; Lake Forest, CA) recorded the data at 120 Hz by tracking infrared light that was reflected from the markers worn by the subjects. The Vicon 524 motion analysis system provided the three-dimensional coordinates of the markers in space and it was then possible to reconstruct the patients' upper body and their upper extremity movements.

### Reconstruction of the Upper Extremities and the Kinematic Model

A bilateral upper extremity model, previously developed by Hingtgen et al [43] and verified for accuracy [44,45], was used to reconstruct the motion of the right and left upper extremities from the VICON data set and to determine the Cartesian joint position and orientation. The model consists of five body segments each of which was defined by at least three non-collinear markers; these were the trunk, the right, and left upper arms and the right and left forearm [43]. These five upper body segments as well as the markers used to define them can be seen in Fig. 2c. The joint centers and joint axes were defined though subject specific anthropometric measurements taken when the subject arrived, as well as marker placement. The position of the joint centers was used as the segment's local coordinate system and the trunk segment was defined with reference to the lab coordinate system. For each task, the markers were manually labeled on the Vicon Body Builder (Vicon, lake for CA), events were marked according to velocity profiles, the data was filtered via a Woltring filter with a predicted mean square error of 20, and then the tasks were loaded for reconstruction.

Our trajectory analysis focused on the Cartesian wrist center data. The model defined the wrist joint center as being located halfway between the radial and ulnar markers according to the following equation (Eq. 1):

w¯c=12(m¯rad+m¯uln⁡)     (Eq. 1)
 MathType@MTEF@5@5@+=feaafiart1ev1aaatCvAUfKttLearuWrP9MDH5MBPbIqV92AaeXatLxBI9gBaebbnrfifHhDYfgasaacH8akY=wiFfYdH8Gipec8Eeeu0xXdbba9frFj0=OqFfea0dXdd9vqai=hGuQ8kuc9pgc9s8qqaq=dirpe0xb9q8qiLsFr0=vr0=vr0dc8meaabaqaciaacaGaaeqabaqabeGadaaakeaacuWG3bWDgaqeamaaBaaaleaacqWGJbWyaeqaaOGaeyypa0ZaaSGaaeaacqaIXaqmaeaacqaIYaGmaaWaaeWaaeaacuWGTbqBgaqeamaaBaaaleaacqWGYbGCcqWGHbqycqWGKbazaeqaaOGaey4kaSIafmyBa0MbaebadaWgaaWcbaGaemyDauNagiiBaWMaeiOBa4gabeaaaOGaayjkaiaawMcaaiaaxMaacaWLjaWaaeWaaeaacqqGfbqrcqqGXbqCcqqGUaGlcqqGGaaicqaIXaqmaiaawIcacaGLPaaaaaa@48AA@

In Equation 1, m¯rad
 MathType@MTEF@5@5@+=feaafiart1ev1aaatCvAUfKttLearuWrP9MDH5MBPbIqV92AaeXatLxBI9gBaebbnrfifHhDYfgasaacH8akY=wiFfYdH8Gipec8Eeeu0xXdbba9frFj0=OqFfea0dXdd9vqai=hGuQ8kuc9pgc9s8qqaq=dirpe0xb9q8qiLsFr0=vr0=vr0dc8meaabaqaciaacaGaaeqabaqabeGadaaakeaacuWGTbqBgaqeamaaBaaaleaacqWGYbGCcqWGHbqycqWGKbazaeqaaaaa@325C@ is the radial marker and m¯uln⁡
 MathType@MTEF@5@5@+=feaafiart1ev1aaatCvAUfKttLearuWrP9MDH5MBPbIqV92AaeXatLxBI9gBaebbnrfifHhDYfgasaacH8akY=wiFfYdH8Gipec8Eeeu0xXdbba9frFj0=OqFfea0dXdd9vqai=hGuQ8kuc9pgc9s8qqaq=dirpe0xb9q8qiLsFr0=vr0=vr0dc8meaabaqaciaacaGaaeqabaqabeGadaaakeaacuWGTbqBgaqeamaaBaaaleaacqWG1bqDcyGGSbaBcqGGUbGBaeqaaaaa@328C@ is the ulnar marker.

### Data Analysis

A custom MATLAB program was used to process and analyze the Cartesian wrist center raw and normalized data corresponding to the desired event(s). Here, we analyze the data for Event 1. Other aspects of the tasks are examined in detailed in Wisneski 2006 [46]. Each reaching event was defined by way of the velocity vector to determine when the subjects started and ended each movement. For example, the reach-to-cup and reach-to-spoon events were defined as beginning at the frame before the initial velocity of the movement and ending at the frame before a change in the velocity vector to indicate the movement of the artifact toward the mouth or bowl.

The Cartesian raw filtered data was used to calculate the following kinematic dependent variables: total displacement (TD), movement time (MT), peak velocity (PV), and movement smoothness (MS). Here, TD is the sum of the raw instantaneous displacements, PV is the maximum velocity value recorded for an event, MT is the total time required to reach the object, and movement smoothness is the number of changes in accelerations within an event.

Normalization of the raw data was performed by subtracting the patient's rest position in Cartesian coordinates from all points along the trajectory, thus all data sets began at (0, 0, 0). The normalized position data were examined for each of the three task conditions. Two key planes of movements (XY and XZ) were analyzed. The XY plane is the horizontal plane of the table and the XZ plane is the vertical plane corresponding to the saggital plane of the torso (Fig. 2). Since subjects moved at their own pace, a 6^th ^order polynomial was used to fit the data for each patient trial and to generate trajectories of the same lengths. The 6^th ^order polynomial was chosen because it was found that it did not compromise or distort the reaching data. The instantaneous tangential velocity was also calculated and plotted for analysis under each condition. Data for the three trials for each subject and all patients in each condition were averaged and resulted in average trajectories for 1–100% of the reach.

The minimum-jerk model was applied next and the resulting curves compared to the average wrist paths generated by the subjects. The context under which the minimum jerk model was calculated was the same as that used to determine point-to-point trajectories in many current robotic therapy environments. Assuming the boundary conditions of zero beginning and ending velocity and acceleration and supplying the initial and final points of the movement in the x, y, and z planes, the 5^th ^order polynomial equations were used to generate predicted trajectories (Eq. 3) [38]:

*x*(*t*) = *x*_*o *_+ (*x*_*o *_- *x*_*f*_)(15*T*^4 ^-6*T*^5 ^- 10*T*^3^)

*y*(*t*) = *y*_*o *_+ (*y*_*o *_- *y*_*f*_)(15*T*^4 ^-6*T*^5 ^- 10*T*^3^)     (Eq. 3)

*z*(*t*) = *z*_*o *_+ (*z*_*o *_- *z*_*f*_)(15*T*^4 ^-6*T*^5 ^- 10*T*^3^)

In Eq. 3, *x*_*o*_, *y*_*o*_, and *z*_*o *_are the starting points of the movements (at *t *= 0) *x*_*f*_, *y*_*f*_, and *z*_*f *_are the final points (at *t *= *t*_*f*_). *T *is scaled as time(t)time(tf)
 MathType@MTEF@5@5@+=feaafiart1ev1aaatCvAUfKttLearuWrP9MDH5MBPbIqV92AaeXatLxBI9gBaebbnrfifHhDYfgasaacH8akY=wiFfYdH8Gipec8Eeeu0xXdbba9frFj0=OqFfea0dXdd9vqai=hGuQ8kuc9pgc9s8qqaq=dirpe0xb9q8qiLsFr0=vr0=vr0dc8meaabaqaciaacaGaaeqabaqabeGadaaakeaadaWcaaqaaiabdsha0jabdMgaPjabd2gaTjabdwgaLjabcIcaOiabdsha0jabcMcaPaqaaiabdsha0jabdMgaPjabd2gaTjabdwgaLjabcIcaOiabdsha0naaBaaaleaacqWGMbGzaeqaaOGaeiykaKcaaaaa@3F91@. Using these equations, we calculated model trajectories for each of the average movement conditions ('present', 'imagined', and 'absent') as well as for all of the individual subject movements in both the XY and XZ planes.

To compare the model and actual trajectories, two metrics were used: the difference in area between curves and curvature. The difference between the averaged and minimum jerk model data was quantified by calculating the area between the curves. The area between curves was calculated for each subject's performance in each condition in both the XY and XZ planes. Since the distance between start and end-points vary, the area was normalized by distance. The curvature was quantified and analyzed using the parametric equations 4 and 5. These equations have been used in many other trajectory analyses and are derived from generalized curvature equations [38,47].

Cxy=x˙y¨−y˙x¨((x˙)2+(y˙)2)32     (Eq. 4)
 MathType@MTEF@5@5@+=feaafiart1ev1aaatCvAUfKttLearuWrP9MDH5MBPbIqV92AaeXatLxBI9gBaebbnrfifHhDYfgasaacH8akY=wiFfYdH8Gipec8Eeeu0xXdbba9frFj0=OqFfea0dXdd9vqai=hGuQ8kuc9pgc9s8qqaq=dirpe0xb9q8qiLsFr0=vr0=vr0dc8meaabaqaciaacaGaaeqabaqabeGadaaakeaacqWGdbWqdaWgaaWcbaGaemiEaGNaemyEaKhabeaakiabg2da9maalaaabaGafmiEaGNbaiaacuWG5bqEgaWaaiabgkHiTiqbdMha5zaacaGafmiEaGNbamaaaeaadaqadaqaamaabmaabaGafmiEaGNbaiaaaiaawIcacaGLPaaadaahaaWcbeqaaiabikdaYaaakiabgUcaRmaabmaabaGafmyEaKNbaiaaaiaawIcacaGLPaaadaahaaWcbeqaaiabikdaYaaaaOGaayjkaiaawMcaamaaCaaaleqabaWaaSGaaeaacqaIZaWmaeaacqaIYaGmaaaaaaaakiaaxMaacaWLjaWaaeWaaeaacqqGfbqrcqqGXbqCcqqGUaGlcqqGGaaicqaI0aanaiaawIcacaGLPaaaaaa@4DE1@

Cxz=x˙z¨−z˙x¨((x˙)2+(z˙)2)32     (Eq. 5)
 MathType@MTEF@5@5@+=feaafiart1ev1aaatCvAUfKttLearuWrP9MDH5MBPbIqV92AaeXatLxBI9gBaebbnrfifHhDYfgasaacH8akY=wiFfYdH8Gipec8Eeeu0xXdbba9frFj0=OqFfea0dXdd9vqai=hGuQ8kuc9pgc9s8qqaq=dirpe0xb9q8qiLsFr0=vr0=vr0dc8meaabaqaciaacaGaaeqabaqabeGadaaakeaacqWGdbWqdaWgaaWcbaGaemiEaGNaemOEaOhabeaakiabg2da9maalaaabaGafmiEaGNbaiaacuWG6bGEgaWaaiabgkHiTiqbdQha6zaacaGafmiEaGNbamaaaeaadaqadaqaamaabmaabaGafmiEaGNbaiaaaiaawIcacaGLPaaadaahaaWcbeqaaiabikdaYaaakiabgUcaRmaabmaabaGafmOEaONbaiaaaiaawIcacaGLPaaadaahaaWcbeqaaiabikdaYaaaaOGaayjkaiaawMcaamaaCaaaleqabaWaaSGaaeaacqaIZaWmaeaacqaIYaGmaaaaaaaakiaaxMaacaWLjaWaaeWaaeaacqqGfbqrcqqGXbqCcqqGUaGlcqqGGaaicqaI1aqnaiaawIcacaGLPaaaaaa@4DEB@

In Eqs. 4 and 5, curvature in the planes are calculated by way of the instantaneous velocities in the x˙
 MathType@MTEF@5@5@+=feaafiart1ev1aaatCvAUfKttLearuWrP9MDH5MBPbIqV92AaeXatLxBI9gBaebbnrfifHhDYfgasaacH8akY=wiFfYdH8Gipec8Eeeu0xXdbba9frFj0=OqFfea0dXdd9vqai=hGuQ8kuc9pgc9s8qqaq=dirpe0xb9q8qiLsFr0=vr0=vr0dc8meaabaqaciaacaGaaeqabaqabeGadaaakeaacuWG4baEgaGaaaaa@2E2E@, y˙
 MathType@MTEF@5@5@+=feaafiart1ev1aaatCvAUfKttLearuWrP9MDH5MBPbIqV92AaeXatLxBI9gBaebbnrfifHhDYfgasaacH8akY=wiFfYdH8Gipec8Eeeu0xXdbba9frFj0=OqFfea0dXdd9vqai=hGuQ8kuc9pgc9s8qqaq=dirpe0xb9q8qiLsFr0=vr0=vr0dc8meaabaqaciaacaGaaeqabaqabeGadaaakeaacuWG5bqEgaGaaaaa@2E30@ and z˙
 MathType@MTEF@5@5@+=feaafiart1ev1aaatCvAUfKttLearuWrP9MDH5MBPbIqV92AaeXatLxBI9gBaebbnrfifHhDYfgasaacH8akY=wiFfYdH8Gipec8Eeeu0xXdbba9frFj0=OqFfea0dXdd9vqai=hGuQ8kuc9pgc9s8qqaq=dirpe0xb9q8qiLsFr0=vr0=vr0dc8meaabaqaciaacaGaaeqabaqabeGadaaakeaacuWG6bGEgaGaaaaa@2E32@ and the instantaneous accelerations x¨
 MathType@MTEF@5@5@+=feaafiart1ev1aaatCvAUfKttLearuWrP9MDH5MBPbIqV92AaeXatLxBI9gBaebbnrfifHhDYfgasaacH8akY=wiFfYdH8Gipec8Eeeu0xXdbba9frFj0=OqFfea0dXdd9vqai=hGuQ8kuc9pgc9s8qqaq=dirpe0xb9q8qiLsFr0=vr0=vr0dc8meaabaqaciaacaGaaeqabaqabeGadaaakeaacuWG4baEgaWaaaaa@2E2F@, y¨
 MathType@MTEF@5@5@+=feaafiart1ev1aaatCvAUfKttLearuWrP9MDH5MBPbIqV92AaeXatLxBI9gBaebbnrfifHhDYfgasaacH8akY=wiFfYdH8Gipec8Eeeu0xXdbba9frFj0=OqFfea0dXdd9vqai=hGuQ8kuc9pgc9s8qqaq=dirpe0xb9q8qiLsFr0=vr0=vr0dc8meaabaqaciaacaGaaeqabaqabeGadaaakeaacuWG5bqEgaWaaaaa@2E31@ and z¨
 MathType@MTEF@5@5@+=feaafiart1ev1aaatCvAUfKttLearuWrP9MDH5MBPbIqV92AaeXatLxBI9gBaebbnrfifHhDYfgasaacH8akY=wiFfYdH8Gipec8Eeeu0xXdbba9frFj0=OqFfea0dXdd9vqai=hGuQ8kuc9pgc9s8qqaq=dirpe0xb9q8qiLsFr0=vr0=vr0dc8meaabaqaciaacaGaaeqabaqabeGadaaakeaacuWG6bGEgaWaaaaa@2E33@. To eliminate any effect due to our polynomial fit procedure, we analyzed the curvature within 5% – 95% of the reach.

### Statistical Analysis

Since the average data for each condition was used for much of the analysis, a repeated measure ANOVA was conducted using MINITAB to determine significant differences both between subject trials and across subjects for all of the comparisons. The condition of task performance (i.e. 'object present', 'object imagined', or 'object absent') as well as subject number were used as terms in the ANOVA model. The subject number was also used as a random factor. For each ANOVA, Tukey's test was used to determine significance between each of the conditions and across subjects if necessary. In order to determine if the heterogeneity in the subject population such as differences in age and arm length affected the results, a repeated measure ANCOVA was conducted using these two variables as covariates.

The dependent variables were also analyzed using the repeated measure ANOVA. When required Tukey's test was performed to determine which conditions produced significantly different dependent variables of movement. We anticipated that the 'object present' condition would show dependent variables reflecting more organized movements, i.e., smooth reaches and shorter times [17-19]. We also expected higher velocities and longer displacements for the object present conditions.

## Results

Table 2 shows the summary values for the drink and feed tasks. When looking at the drink task, it can be seen that there is not a statistically significant difference between the total displacements, the movement times, or the movement smoothness for any of the three conditions. The peak velocity for the object present condition is statistically greater than for the other two conditions and the peak velocity for the object absent condition is statistically greater than for the object imagined condition.

**Table 2:** Averaged Movement Dependent Variables for each condition.

**DRINK**	**Present**	**Imagined**	**Absent**	**ANOVA (p-value)**	**Tukey's Test**
TD (mm)	**350 **+/- .06	**400 **+/- .04	**320 **+/- .05	.009	I > P & A
MT(sec.)	**1.02**+/- .12	**.94 **+/- .09	**.96 **+/- .1	.334	=
PV (mm/s)	**893.5 **+/- 14	**754.8 **+/- 11	**781.6 **+/- 8.9	< .0001	P > I & A
MS (+/- uts)	**3 **+/- .25	**3 **+/- .25	**3 **+/- .5	.99	=
**FEED**					
TD (mm)	**250 **+/- .02	**230**+/- .01	**230 **+/- .01	.008	P > I & A
MT (sec.)	**.78 **+/- .1	**.82 **+/- .11	**.77 **+/- .08	.35	=
PV (mm/s)	**752 **+/- 9	**788 **+/- 8.8	**761 **+/- 9.5	< .001	I > P & A
MS (+/- uts)	**2 **+/- .2	**2 **+/- .4	**2 **+/- .25	.89	=

The average values for Total Displacement (TD), Movement Time (MT), Peak Velocity (PV), and Movement Smoothness (MS) for all three conditions. The value for the repeated measure ANOVA and the ordering results from Tukey's test can be found in the last two columns

The data for the feed task shows that again there is no statistically significant difference for total displacement, movement time, or movement smoothness between any of the three conditions. In the case of the peak velocity, the object imagined condition shows significantly greater peak velocity than the object present and absent conditions do which are not significantly different from each other. It is possible that we do not see the same pattern in peak velocity as for the drink task due to the fact that all three conditions of this task were much more similar to each other and resembled a point-to-point reach more closely than the drink task did. This may have caused the three conditions to be performed more similarly in dependent variables.

### The Drink Task

**Figure 3:**
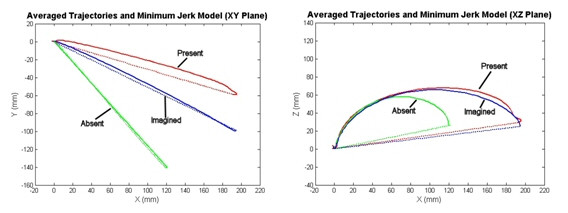
**Reaching-to-Cup Event Cartesian data and Minimum Jerk Model in XY and XZ Planes**. Left: XY plane averages of the drink task in all three conditions plotted with the minimum jerk model for the movements. (ND drink (middle, red), Drink Imagined (top, blue), Drink Absent (bottom, green)). Right: XZ plane averages of the drink task in all three conditions plotted with the minimum jerk model for the movements. (ND drink (top, red), Drink Imagined (middle, blue), Drink Absent (bottom, green)).

The averaged trajectories for the reach-to-cup event of the drink task plotted with the minimum jerk models for each condition can be seen in figure 3. We anticipated that the 'object absent' condition would fit the predicted minimum jerk model the best since it is most similar to the point-to-point conditions that were observed when the model was developed. We also anticipated that the 'object present' condition would show the most deviations caused by the presence of the functional objects.

It is clear from Figure 3 (left) that the model in the XY plane does fit the 'object absent' (point-to-point) condition the best. The 'object present' condition produces the trajectory that shows the most deviations from the minimum jerk model data.

In contrast, the trajectories in the XZ plane do not fit the model data in any of the three conditions. All conditions produce a movement with a similar curvature pattern that shows a large displacement in the Z (vertical) direction. The curvature seen in this plane is thought to be a result of the subjects reaching in an 'up-and-over' fashion to work around the table constraint due to the starting location and position of their hand. Since the subjects begin the tasks with their palms face down on the table it may have been natural to lift the hand as the movement occurs to be sure the hand clears the table as the goal is approached.

### The Feed Task

**Figure 4:**
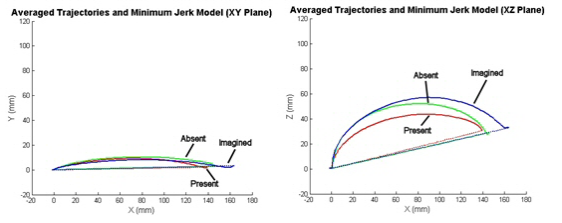
**Reaching-to-Spoon Event Cartesian data and Minimum Jerk Model in XY and XZ Planes**. Left: XY plane averages of the feed task in all three conditions plotted with the minimum jerk model for the movements. (ND drink (red), Drink Imagined (blue), Drink Absent (green)). The trajectories in the XY plane for the reach-to-spoon event are not statistically significantly different. Right: XZ plane averages of the feed task in all three conditions plotted with the minimum jerk model for the movements. (ND drink (bottom, red), Drink Imagined (top, blue), Drink Absent (middle, green))

To investigate whether object location and affordance contributed to the deviations we observed in the drink task, we examined the reach-to-spoon component of the feed task. We anticipated that similar patterns of movement would be seen across tasks, however, the orientation and placement of the object would cause some curvature differences in the trajectory. The average trajectories and corresponding minimum jerk models for all three conditions of the feed task in the XY and XZ planes can be seen in figure 4.

The XY data in figure 4 (left) shows that the trajectories for the reach-to-spoon task in all three conditions are not statistically significantly different from each other (p = 0.405), which is not what was seen in the drink task where the 'object present' condition produced movements with greater curved deviations. The XZ data in figure 4 (right) shows that there is a similar curvature pattern and deviation in the positive Z direction to that seen in the drink task. This again is thought to be due to the table constraint.

### Velocity for feed and drink tasks

**Figure 5:**
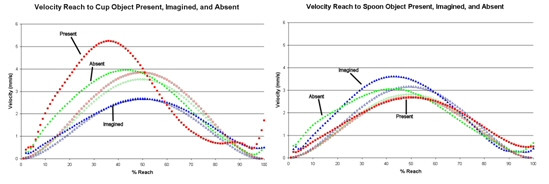
**Velocity Profiles**. Velocity profiles for reach-to-cup (Left) and reach-to-spoon (Right) events in all three conditions as well as profiles for the corresponding minimum jerk models. (Present condition is represented by squares; imagined condition is represented by triangles; and absent condition is represented by circles. All averaged data is filled and all minimum jerk model data is not filled.)

As stated previously, the velocity of the reaching movements is also an important aspect of movement to analyze. The minimum jerk theory of movement predicts that the straight line movements will have bell shaped velocity profiles with zero velocity at the beginning and ending of the movements. In order to determine if this constraint held true for functional movements the instantaneous velocity for the averaged trajectories in each condition was plotted for both the reach-to-cup and reach-to-spoon events (Fig. 5).

As can be seen in figure 5, the velocities for all of the minimum jerk model data are symmetric bell shaped profiles with 0 beginning and ending velocity as expected. A salient feature across both tasks is that unlike the model data, the actual data does not show subjects ending the reach at zero velocity.

The velocity profiles for the reach-to-spoon event reach a peak of a lesser magnitude than those of the reach-to-cup event (p < 0.001). This is expected since the distance travelled to the cup was greater than that travelled to the spoon (Table 2). The 'object present' condition of the drink task produced the greatest peak velocity.

Another point of interest between tasks is that the velocity profiles for the reach-to-spoon task are more symmetric (peak at 50.35 +/- 8%) and closer to the minimum jerk prediction than the velocity profiles for the Reach-to-cup task (peak at 37.75 +/- 1.5%).

#### Differences between Model and Reach-to-Cup and Reach-to-Spoon

In order to quantify some of the differences seen between averaged data and what the model predicts, two metrics were looked at; the area between curves and the curvature of the paths.

##### Area Deviations

**Figure 6:**
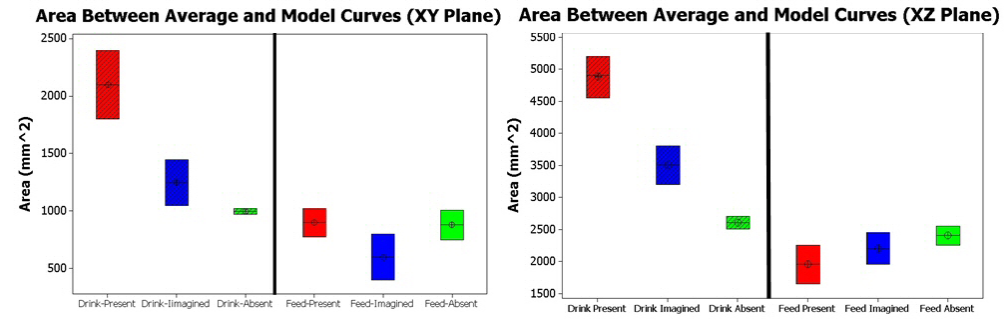
**Area between Average and Minimum Jerk Curves in XY and Planes**. Left: Area between the model curve and the normalized curves for paths in the XY plane. (In order from left to right ND Drink (red), Drink Imagined (blue), Drink Absent (green), ND Feed (red), Feed Imagined (blue), Feed Absent (Green). Average area in the XY plane is 1121.7 mm^2. The object present condition of the drink task produces significantly greater curvature than the other two conditions (p < .0001), which are not significantly different from each other. The feed task shows no significant differences between any of the three conditions. Right: Area between the model curve and the normalized curves for paths in the XZ plane. (In order from left to right ND drink(red), Drink Imagined (blue), Drink Absent (green), ND Feed (red), ND Drink (blue), ND Absent (green). Average area in the XZ plane is 2922.2 mm^2. The object present condition of the reach-to-cup event of the drink task has the greatest area (p < .0001).

Figure 6 shows the area between curves for both tasks as well as in all three conditions.

Initially it can be seen that the areas in the XZ plane for both the drink and feed tasks are more than double the areas in the XY plane (XY average = 1121 mm^2^, XZ average = 2922 mm^2^) (Fig. 6). These results show that movements in the XZ plane produced more curvature deviations than those in the XY plane.

##### Curvature

**Figure 7:**
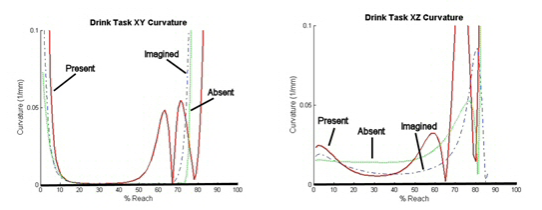
**Curvature in the XY and XZ Planes**. Left: Curvature in the XY plane plotted against % Reach. Average minimal curvature = .0007/mm. Right: Curvature in the XZ plane plotted against % Reach. Average minimal curvature = .0031/mm.

The curvature plots for the reaching event of the drink and feed tasks in the XY and XZ planes can be seen in Figure 7.

In this figure, the first feature to note is that for all movements the maximal curvature is at the beginning and end of the movements. Another interesting feature can be noted at the curvature minima. In the XZ plane, the average minimal curvatures deviate more from 0 than in the XY plane (XZ average: 0.0031/mm > XY average: 0.0007 (p < .001). This shows that at the minimal curvature, the movement in the XZ plane shows the greatest deviations from the minimum jerk trajectory, which is 0, in all three conditions. This will be important information in the development of a functional trajectory model.

The XY plane data (Fig. 6) for the drink task shows that the object present condition produces significantly greater curvature than the object absent and imagined conditions (p < 0.05), which are not significantly different from each other (p = 0.087). This shows that the addition of a functional object, in this case a cup, creates deviations from the minimum jerk trajectory that are not otherwise seen. The object absent condition produces trajectories that are the most repeatable in their resemblance to the minimum jerk model data as can be seen by the small standard deviation.

The XZ plane data for the drink task reveals a similar pattern. Again the object present condition produced a trajectory with the greatest deviation. The object imagined condition produced a trajectory with greater deviation than the object absent condition in the XZ plane. This may have been occurred because the subject was imagining a handle of a cup that was raised from the table top, thus requiring a different approach in the vertical direction than for the object absent condition.

Looking at the feed task in the XY plane it can be seen that there is no statistical difference between any of the three conditions. This shows that the orientation required for grasping and manipulating the spoon in this location required a wrist trajectory similar to that required to point to the same location. All three feed conditions in the XY and XZ planes show a significantly lesser area than those for the drink task. This indicates that the trajectories generated in the feed task follow more closely to the minimum jerk model than those for the drink task. This highlights the importance of object affordance and placement. The differences seen between the drink and feed task indicate that a functional model must accommodate a wide range of object affordances in order to be adequate to implement in the robotic therapy environment.

Finally, when a repeated measure ANOVA was conducted it was found that there were not interactions across subjects or trials within each condition. The results for the repeated measures ANCOVA showed that the age and arm length of the subjects did not significantly affect the wrists paths. Thus, the averaged data is used in the subsequent analysis.

## Discussion

Through the data presented here we have highlighted concerns that must be addressed when modeling functional movements. We have shown that as object affordance changes there are significant differences in the kinematics, specifically in the curvature of movements performed. These findings are important to consider when implementing trajectories in robotic therapy environments for stroke rehabilitation. Our results agree with previous research, which has shown that object affordance and functional goal performance can influence therapy outcomes [17-19]. These task-dependent, functional improvements are important when planning therapy options for stroke survivors because of the influence that functional task practice may have on brain plasticity [48-53]. Many previous studies have shown that it is important to support functional activities in therapy environments in order to achieve optimal recovery [50-53].

The data we have presented indicates that in order to support these functional tasks in our robotic therapy environment it is necessary to accommodate the curvature deviations from the minimum jerk model seen in these trajectories. The major differences that we have highlighted include trajectory dependence on: object presence, object orientation, and the plane of movement.

We have shown that when subjects reach to a point in space or an imaginary object, their trajectories adhere more closely to the predicted minimum jerk model than when there is an actual object present (Figs. 3 and 4). Studies have shown that when subjects are performing functional reaching to real-life objects the orientation of the hand and the grasp aperture is adjusted to accommodate for object orientation, shape, and size [54-57]. We looked at both the XY and XZ data for reaches to a cup and a spoon. In the XY plane, our data show that when the subjects reached to the cup, versus a point in space, their trajectories became more curved (Fig. 3). This is thought to be due to the hand orientation for grasping and stabilization requirements of the functional task. Grasping and orientation has been a topic of much previous research [54-63]. It has been shown that the orientation of the hand when approaching an object depends on many variables including object shape, size, orientation, location, and properties (such as weight and friction characteristics) [55,58-63].

Comparatively, this did not apply to reaches to a spoon. In the case of the spoon, the trajectories in the XY plane were not significantly different between conditions. This is thought to be because the location of the spoon on the table and the hand orientation required to grasp the spoon were similar to the requirements of the point-to-point version of the task. Thus all three conditions resulted in more similar movements than seen in the drink task where when the subjects were required grasp a cup, their trajectory became more curved.

The XZ trajectories show similar patterns across the drink and feed tasks (Figs. 3 and 4). Vertical (Z-direction) deviations can be seen for these reaches to both objects. These deviations are thought to be due to the avoidance of the table constraint. The fact that the same pattern is seen in the XZ plane for two tasks requiring different hand orientation and positioning indicates that this may be an important feature of movement to be considered for the functional model. The differences in the XY plane data indicate trajectory dependence on object location and orientation. From this analysis it can be concluded that it will be important for the model to both capture patterns of movements that are invariant across tasks as well as react differently to various object and task specific requirements.

The data from the velocity profiles and from the dependent variables support the Cartesian wrist trajectory findings. The 'object present' condition of the drink task shows the greatest peak velocity (Table 2 and Fig. 5). The fact that the peak velocity is greatest when there is a functional object present agrees with the data collected by Trombly, Wu, and colleagues which shows that object affordance leads to movements with improved dependent variables including higher peak velocities [17-19]. This does not apply in the case of the feed task however. Just as in the Cartesian data we see a closer resemblance to the point-to-point version of the task. This is thought to be due to the object affordance. The spoon was laying flat on the table and required the subjects to perform a movement more closely resembling a point-to-point reach. The cup required the subjects to reach out of the plane of the table to the handle, thus deviating from planar point-to-point movements more. This again supports the need for the model to account for various task specific requirements rather than approaching all functional tasks in the same way.

Finally the curvature analysis also agrees with these findings as we see that the drink task produces greater area between curves than the feed task (Fig. 6). In addition, when looking at the drink task, that the XZ plane shows greater minimal curvature than the XY plane (Fig. 7). This brings an important point for modeling considerations to light. The functional model that is developed must account for this increased curvature seen in the XZ plane, while providing significantly less curvature in the XY plane.

This analysis has helped us to see that trajectory dependence on object presence, orientation, and plane of movement, will prove to be critical in developing a functional model. Supporting highly functional activities via a robot-assisted environment will require a trajectory model that considers these object affordances and the influence of the plane of movement on the trajectory paths.

### Sources of Error and Other Considerations

The data we have presented here comes from a small sample (8 subjects) the question of how this sample will generalize to the population must be considered. However, we do see a high level of repeatability between subjects indicating that the patterns we observe are characteristic of natural movements. We will have to investigate more tasks and more task types to determine how various objects, object placements, and goals affect trajectories.

The constraints that we put on the tasks to control the data set may not account for all natural movement settings. We required the subjects' hands to start and end each task in a prescribed rest position. This positioning of the hand may have affected the reach trajectory. Other rest positions should be considered.

Variability between trials and subjects could also be a source of error for our data set. Objects may have been moved slightly between trials. And the point-to-point location prescribed did not have a visual cue so subject may have reached to different locations for each trial.

Finally, since our goal is to implement this trajectory model on a stroke therapy system, we must consider how the trajectories may need to be adjusted for stroke survivors. We expect to see movements with lower peak velocities, increased total displacements, and decreased smoothness. We expect that the velocity profiles will have multiple peaks to account for adjustments made in movement direction. These and other factors must be studied in order to make this mode appropriate for stroke survivors.

## Conclusion

The data presented in this paper confirms that a new approach is necessary when implementing functional tasks in the ADLER therapy environment. From this data we have learned that we not only have to be concerned with the transport component of the reach and moving the patients hands to the proper location, but also with the orientation component of the reach and positioning their hands properly to grasp the object. Since the orientation component seems to be captured in the increased curvature of the trajectory this will be the focus of the model we are creating.

In the drink and feed tasks from the previous analysis, only two objects were analyzed; the cup and the spoon. These objects were of different shape, size, orientation, and location on the activity table. The fact that all of these variables were varied at the same time, does not allow for individual causes of path shape difference to be determined. A future experiment that specifically examines the effect on the wrist path of individually varying each of these variables in order to determine which variables cause what type of curvature deviations would be very valuable. Many more object types should be examined as well as more object locations. With this information it would be possible to update the model so that inputs could include object location, orientation, size, and shape and result in more appropriate path generation.

In the future, we will explore the patterns in curvature and velocity that emerge between sets of like movements within tasks for complete tasks. We will look at the other paradigms for implementing the minimum jerk theory of movement including the via-point application to help in the development of a new model for implementation in ADLER. We will also investigate the differences seen between stroke patients' and normal, healthy subjects' movements to determine what adaptations to the model are necessary. We will then implement this model on the ADLER environment.

By developing a model that will allow the execution of more natural movements in their rehabilitation we hope to create an environment that affords patients reliable and repeatable practice of activities of daily living. When patients are trained with functional task specific movements using natural trajectory patterns we hope to elicit brain plasticity to encode for these natural movements. Through their practice of daily living exercises we hope to see gains made in their movement kinematics and increases in their functional ability. We also hope to increase patients' confidence in executing real-life tasks with their impaired arm so that carryover is increased.

## Competing interests

The author(s) declare that they have no competing interests.

## Authors' contributions

KJW and MJJ were involved in all stages of subject recruitment and data acquisition. KJW was the primary composer of the manuscript with major contributions by MJJ. MJJ generated the initial concept for the studies and oversaw the progress and analysis. All authors contributed significantly to the intellectual content of the manuscript and have given final approval of the version to be published.
